# Large increase in fracture resistance of stishovite with crack extension less than one micrometer

**DOI:** 10.1038/srep10993

**Published:** 2015-06-08

**Authors:** Kimiko Yoshida, Fumihiro Wakai, Norimasa Nishiyama, Risako Sekine, Yutaka Shinoda, Takashi Akatsu, Takashi Nagoshi, Masato Sone

**Affiliations:** 1 Secure Materials Center, Materials and Structures Laboratory, Tokyo Institute of Technology, R3-23 4259 Nagatsuta, Midori, Yokohama, 226-8503, Japan; 2 Deutsches Elektronen-Synchrotron (DESY), Notkestr. 85, 22607 Hamburg, Germany; 3 Precursory Research for Embryonic Science and Technology (PRESTO), Japan Science and Technology Agency (JST), Chiyoda, Tokyo 102-0075, Japan; 4 Precision and Intelligence Laboratory, Tokyo Institute of Technology, R2-35 4259 Nagatsuta, Midori, Yokohama, 226-8503, Japan

## Abstract

The development of strong, tough, and damage-tolerant ceramics requires nano/microstructure design to utilize toughening mechanisms operating at different length scales. The toughening mechanisms so far known are effective in micro-scale, then, they require the crack extension of more than a few micrometers to increase the fracture resistance. Here, we developed a micro-mechanical test method using micro-cantilever beam specimens to determine the very early part of resistance-curve of nanocrystalline SiO_2_ stishovite, which exhibited fracture-induced amorphization. We revealed that this novel toughening mechanism was effective even at length scale of nanometer due to narrow transformation zone width of a few tens of nanometers and large dilatational strain (from 60 to 95%) associated with the transition of crystal to amorphous state. This testing method will be a powerful tool to search for toughening mechanisms that may operate at nanoscale for attaining both reliability and strength of structural materials.

The enhancement of reliability and damage tolerance are the critical issues for the development of high performance ceramics, which are hard and wear-resistant, but, brittle in nature. The fracture resistance of ceramic materials is increased with crack extension by toughening mechanisms^1^ such as crack bridging^2^, microcracking, or phase transformation^3^,^4^. Materials with rising resistance-curve (R-curve) lead to an increase in the strength compared to a material with the same intrinsic toughness and a flat R-curve^5^. The R-curves can be measured by using specimens with macrocracks (mm size) or with microcracks (~100 μm size). R-curves depend on specimen geometry, and they are not materials constants^6^. But, the early part of the R-curve, which is relatively insensitive to specimen geometry, provides the information on factors related to microscopic toughening mechanisms. The evaluation of R-curves with crack extension less than 10 μm is critical for the design of ceramic microstructures with both high strength and high toughness, because the allowable flaw size is small in ceramics^7^,^8^,^9^.

We propose a method to investigate R-curve behavior by using micro-cantilever beam, which has been originally used to measure fracture toughness of amorphous/glass^10^, thin films^11^, single crystals^12^, and grain boundaries^13^. The micro-cantilever beam technique is applied to study the microscopic crack shielding mechanisms of nanocrystalline stishovite ceramics, which exhibit a large fracture toughness due to fracture-induced amorphization^14^,^15^. Stishovite is a high-pressure phase of silicon dioxide (SiO_2_) stable at pressures above 9 GPa, and metastable at ambient conditions^16^. While low-pressure phases, e.g., quartz and coesite, consist of networks of corner-sharing SiO_4_ tetrahedra, stishovite possesses the rutile structure and is described as chains of edge-sharing SiO_6_ octahedra. Stishovite is 60% more compact than quartz, and has the highest hardness (33 GPa) of any stable/metastable oxide under ambient temperatures^17^. Only diamond and cubic boron nitride are industrially used materials that are harder than stishovite. Hard materials with limited ability of plastic deformation tend to be brittle, then, the fracture toughness *K*_*1C*_ of a single crystal is 1.6 ± 0.3 MPa m^1/2^ by the indentation method^18^. Recently, Nishiyama^14^ found that a polycrystalline stishovite with submicron grain size had a fracture toughness of 7.9 MPa m^1/2^ by the indentation fracture method (IF method). Furthermore, a nanocrystalline stishovite with the average grain size of 127 nm had fracture toughness of 13 ± 3 MPa m^1/2^. The IF method is highly questionable for estimating the accurate fracture toughness value^19^, but, the results indicate the fracture toughness increases with decreasing grain size. This discovery shows that the nanocrystalline stishovite attains both excellent hardness and toughness which are vital requirements for structural materials.

Transformation of metastable stishovite with six-fold coordination to the stable phase with four-fold coordination often occurs through intermediate amorphous state, because the Gibbs free energy of stishovite is higher than that of amorphous SiO_2_ at any temperature above 0 K at ambient pressure^20^. The direct transition of crystal to amorphous state, which is analogous to melting, is triggered by heating at 1 bar, or decompression at room temperature. The terms, amorphization/vitrification and melting are used for the transition below and above the glass transition temperature. The amorphization accompanies a volume expansion up to about 100%. The amorphization is induced by fracture since the tensile stress, which is equivalent to negative pressure, can be very large at the crack tip. The formation of amorphous phase with the thickness of a few tens of nanometers was observed on the fracture surface by X-ray absorption near edge structure (XANES) spectroscopy^15^. In analogy to the transformation toughening of zirconia where metastable tetragonal phase transforms to stable monoclinic phase at crack tip^3^,^4^, the fracture-induced amorphization can be a toughening mechanism of stishovite. In order to understand why the nanometer-thick amorphous layer can increase the fracture resistance significantly, we have investigated R-curve behavior by using micro-cantilever beam specimens.

## Results

**Figure 1:**
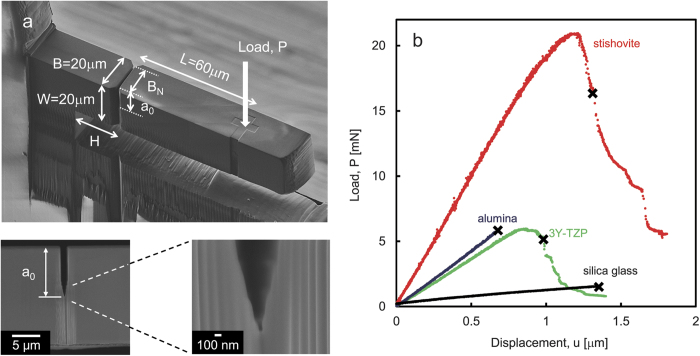
Micro-mechanical testing. (**a**) Geometry of a micro-cantilever beam specimen. The side-grooves were cut to guide the crack. Lower parts show the side view of the notch. (**b**) Load-displacement curves (P – u curves).

**Figure 2:**
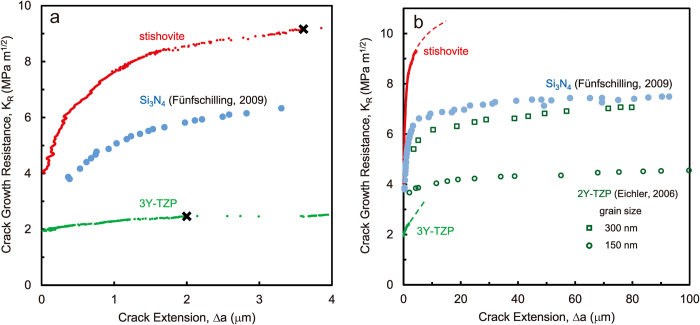
R-curve measurements. (**a**) Microscopic R-curve behavior of nanocrystalline stishovite and zirconia (Y-TZP). (**b**) Macroscopic R-curve behaviors.

The nanocrystalline stishovite with the average grain size of 128 nm was synthesized at 1473 K and at a pressure of 15 GPa by using a pure bulk silica glass as the starting material^15^. For comparison, we have also studied a pure silica (SiO_2_) glass, a submicron alumina (Al_2_O_3_), and 3 mol%-Y_2_O_3_ stabilized tetragonal zirconia polycrystals (ZrO_2_, 3Y-TZP). The full detail of materials and specimen preparations are included in the supplementary information. The micro-cantilever beam specimens with notch were used to measure R-curve behavior in microscopic scale (Fig. 1a). The resulting load (P) – displacement (u) curves are plotted in Fig. 1b. Unstable fracture occurs when the material has a flat R-curve. The principal mechanism of rising crack resistance in alumina is grain-localized bridging, which is more pronounced for coarse-grained materials than for fine-grained materials^21^, then, the R-curve of fine-grained alumina is almost flat. As expected, pure silica glass and the alumina with submicrometer grain size showed unstable fracture. The stress intensity factor *K*_0_ at the crack initiation was 0.8 MPa m^1/2^ for pure silica glass, and 2.8 MPa m^1/2^ for the alumina. The measured values were in good agreement with the fracture toughness reported by macroscopic tests. In the materials which have rising R-curves, the crack starts propagation at *K*_0_, and grows stably, and then, unstable fracture occurs at a critical crack extension^5^. The stable fracture was observed in 3Y-TZP and stishovite. The occurrence of unstable fracture is indicated by the “x” mark. The R-curves were calculated by the compliance method from P-u curves, and plotted in Fig. 2a. The dimensions of each specimen and elastic constants used in the analysis are summarized in Supplementary table S1. The crack growth resistance of stishovite increased from *K*_0_ = 4 to 8 MPa m^1/2^ with crack extension about one micrometer. The initial slope Δ*K*/Δ*a* was 7 MPa m^1/2^/μm at *K*_0_. In 3Y-TZP, the crack growth resistance increased from *K*_0_ = 2 MPa m^1/2^ with the initial slope of 0.2 MPa m^1/2^/μm. The initial steep rise in toughness has been found also in the silicon nitride (Si_3_N_4_) with MgO and Y_2_O_3_ as sintering aids by correcting the effect of notch root radius of macroscopic specimens^8^. The initial slope of stishovite was more than three times steeper than that of the silicon nitride toughened by bridging mechanisms^9^. The R-curves measured by micro-cantilever beam specimens show only the early part of rising R-curve, and cannot be used to determine the plateau value. But, they are advantageous to determine *K*_0_ and the initial slope accurately. R-curve behaviors with crack extension of several tens of micrometers are shown for some ceramics in Fig. 2b. The extrapolation of R-curve of stishovite indicates that it can reach the stress intensity factor higher than 10 MPa m^1/2^, which is close to the fracture toughness measured by IF method. The plateau value of nanocrystalline stishovite is higher than that of the silicon nitride^8^ and 2 mol%-Y2O3 stabilized tetragonal zirconia polycrystals (2Y-TZP)^22^. The fracture toughness of nanocrystalline Y-TZP materials increases with increasing grain size and decreasing dopant content. The fracture resistance of 3Y-TZP measured in air was lower than that of 2Y-TZP measured in vacuum due to the susceptibility to subcritical crack growth under humid atmosphere.

**Figure 3:**
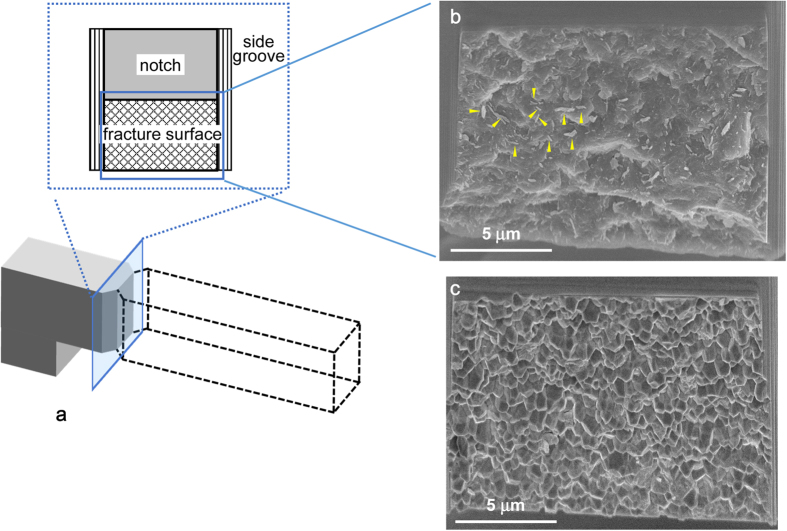
Fracture surface of micro-cantilever beam specimen. (**a**) Specimen geometry. (**b**) nanocrystalline stishovite. Arrows show some examples of “worms”. (**c**) Y-TZP.

Figure 3 shows SEM micrographs of fracture surfaces of nanocrystalline stishovite (Fig. 3b) and Y-TZP (Fig. 3c). The fracture surface of the nanocrystalline stishovite showed a rough morphology accompanied with many worm-like textures scattered over the whole surface. The dimensions of a “worm” are several tens and hundreds of nanometers in diameter and length, respectively. This feature of nanocrystalline stishovite was quite different from that of the intergranular fracture of Y-TZP. The worm-like textures observed on a micro-cantilever beam specimen of nanocrystalline stishovite had been also observed on a surface of mechanically crushed sample^15^. The selected-area electron diffraction patters of the worm-like textures had a halo, then, it showed the presence of amorphous phase on the fracture surface^15^.

## Discussion

**Figure 4:**
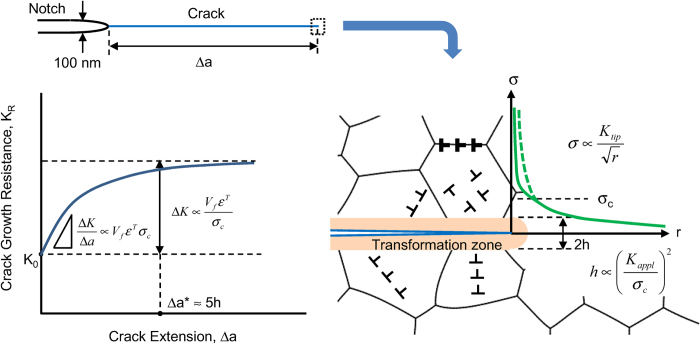
R-curve behavior by transformation toughening. The crack-tip stress field is altered by the formation of transformation zone at the critical stress *σ*_*c*_. The stress intensity change Δ*K* by the presence of transformation zone is the origin of the transformation toughening. The amorphization of nanocrystalline stishovite is supposed to nucleate at surfaces, grain boundaries, and dislocations near the crack tip.

The effect of extrinsic toughening mechanism is described by a stress intensity change Δ*K* between the applied stress intensity factor and the stress intensity factor at the crack tip, which is assumed to be equal to the intrinsic toughness *K*_0_ of the material. In the transformation toughening, the transformation zone develops upon crack extension by stress-induced transformation as illustrated in Fig. 4^3^,^4^. The transformation zone width *h* depends on the critical stress *σ*_*c*_ for the phase transformation:


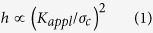


The transformation zone width increases with crack extension Δ*a*, and then, saturates at Δa* ≈ 5*h*. The extent of Δ*K* is proportional to 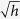 and the dilatational strain *ε*^*T*^ accompanying transformation (i.e. the volume increase by the transformation):


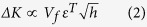


where *V*_*f*_ is the volume fraction of transformed phase in the transformation zone. From equations (1) and (2), the toughness is expressed as:


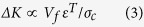


The sharply rising R-curve indicates that the transformation zone width *h* of stishovite is much narrower than that of Y-TZP. This is the evidence that the toughening by fracture-induced amorphization acts in nano- and submicron- scales. The width *h* of Y-TZP is estimated from the monoclinic phase content measured by Raman microscopy: 4 μm for the grain size of 900 nm, and 2 μm for the grain size of 140 nm^22^. The critical stress *σ*_*c*_ for transformation determines the transformation zone width *h*. *σ*_*c*_ is estimated to be about 1.3 GPa for tetragonal-monoclinic transition in 2Y-TZP^23^.

As well as stishovite, dense high pressure crystalline silicates, where silicon is in octahedral coordination, transform under tension to a low-density glass composed of tetrahedral silicon. The toughening by fracture-induced amorphization can occur in a number of such materials, for example, CaSiO_3_ and MgSiO_3_ perovskites. In the molecular dynamic simulation, high-pressure phases of CaSiO_3_ and MgSiO_3_ started amorphization at tensile stress of 18 GPa at 300 K^24^. This result suggests that the critical transformation stress of stishovite is more than 10 times higher than that of Y-TZP. From equation (1), the transformation zone width of stishovite is estimated to be a few tens of nanometers. This estimate agrees with the experimental observation by the surface-sensitive Si-K XANES measurement^15^.

The initial slope of R-curve Δ*K*/Δ*a* is a function of parameter 
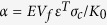
, and increases linearly with *α* approximately^25^. While the fracture toughness of stishovite was about two times larger than that of Y-TZP, Δ*K*/Δ*a* of stishovite was 30 times larger than that of Y-TZP. The slope of stishovite is very steep, because it is proportional to the product of *ε*^*T*^ and *σ*_*c*_, both of which are about 10 times larger in stishovite than in Y-TZP. Steep initial slopes are desirable for R-curves because materials that achieve high toughness at small crack length also achieve high strength.

The increase of fracture toughness with decreasing grain size of stishovite is opposite to the behavior expected from the crack bridging effect and the results observed in transformation toughening of Y-TZP^26^. But, the grain size effect on fracture toughness cannot be separated from other factors such as internal microstrain and dislocation densities^14^. The microstrain arose during synthesis of stishovite which crystallized from bulk glass at 15 GPa. As the synthesis temperature decreases, the grain size decreases, and the microstrain increases.

The amorphization of stishovite is dominantly a heterogeneous nucleation and growth controlled process^27^,^28^. The transformation proceeds by nucleation of amorphous at defects, such as grain boundaries, surfaces, and dislocations^27^. Many dislocations were observed in fine stishovite grains by TEM observation^11^. The origin of dislocations is unknown. One possible explanation is that local deformation occurred during the phase transformation from glass to stishovite at the synthesis pressure^29^. Dislocation density decreased with increasing the synthesis temperature, and then, grain size. It is possible that the fine grain size promotes the amorphization process due to high defect density. Indeed the amount of transformed phase on the fracture surface and the fracture toughness increased with decreasing grain size^15^. The high density of nucleation sites increases the volume fraction of amorphous phase in the transformation zone, which in turn, enhances the crack shielding effect Δ*K* in equation (3).

**Figure 5:**
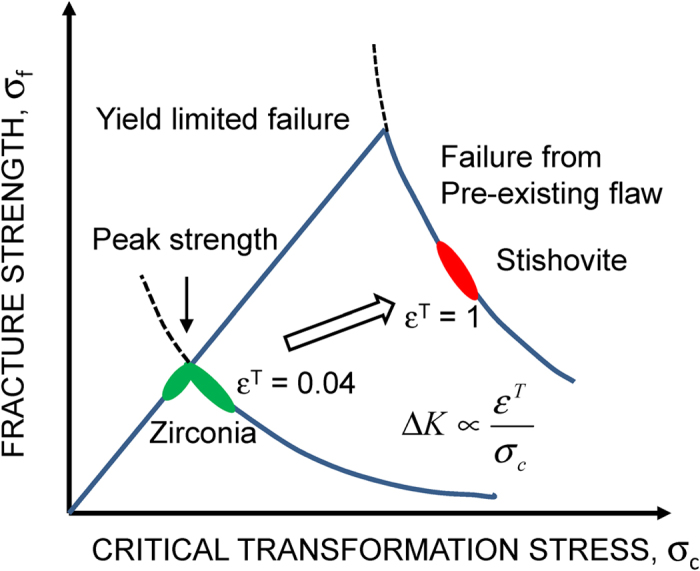
Relation between strength and critical transformation stress *σ*_*c*_. The strength is limited by fracture toughness in the failure from pre-existing flaw region. The strength is limited by *σ*_*c*_ in the yield limited region.

The relation between fracture strength and the critical transformation stress is illustrated in Fig. 5. When the strength is limited by failure from pre-existing flaws, the strength is proportional to fracture toughness. From equation (3), the toughness, and then, strength increase as *σ*_*c*_ decreases. However, as the critical stress decreases, the strength is limited by *σ*_*c*_ due to the transformation induced deformation^4^,^30^. The achievement of peak strength by optimizing *σ*_*c*_ is the target in the material design of zirconia. In fracture-induced amorphization of stishovite, the volumetric transformation strain *ε*^*T*^ is from 60 to 95%, while it is about 4% in the tetragonal-monoclinic transition of zirconia. From equation (3), the increase of *ε*^*T*^ enhances the fracture toughness at a given *σ*_*c*_ as illustrated in Fig. 5. This effect shows that the peak strength of stishovite can be higher than that of zirconia ceramics. The very narrow transformation width and high critical stress are effective in toughening of stishovite due to large *ε*^*T*^.

In summary, the fracture resistance measurement by using micro-cantilever beam specimens revealed that there exists a toughening mechanism which can operate at nano-scale. The narrow transformation zone in fracture-induced amorphization of nanocrystalline stishovite was very effective to increase the fracture resistance by a crack extension of less than one micrometer. This finding will provide a motivation to search for other nano-scale toughening mechanisms in many structural materials, such as ceramics, composites, nanocrystalline materials, and also, synthetic materials with hierarchical architecture.

## Methods

### Material

Nanocrystalline stishovite samples were synthesized using a Kawai-type multi anvil apparatus^15^. The dimensions of the starting material (pure SiO_2_ glass) are 2.5 mm in diameter and 1.4 mm in height. Load was applied first up to 15 GPa under room temperature, and the temperature was increased rapidly from ∼450 °C to 1473 K within 30 s at constant load. The temperature was held constant for 30 min. The decompression (3 h) was started when the temperature was decreased to ∼450 °C. The nanocrystalline stishovite contained numerous dislocations.

### Mechanical testing

The loading of the cantilever beams is carried out in air using a mechanical testing machine designed for micro-sized specimen. The basic concept of this machine is described in ref. 10. The displacement was applied by a piezoelectric device at a displacement rate of 10 nm/sec. The load was applied by using a spherical diamond indenter with radius of 2.5 μm. The machine compliance was 0.023 μm/mN. Load and displacement data were acquired with the sampling interval of 0.1 second.

### Stress intensity factor and compliance of micro-cantilever beam specimen

The stress intensity factor (mode I) *K*_*I*_ of a side-grooved cantilever beam specimen in Fig. 1a was calculated by the extended finite element method (XFEM) using the commercial solver Abaqus 6.13. The stress intensity factors were determined by path-independent contour integral. Side grooves were introduced for straight crack propagation under the presence of *K*_*II*_ component in cantilever beam test. Three-dimensional isotropic elastic model was employed to simulate the cantilever beam specimens with side grooves (*H*: *L*: *W*: *B* = 1:3:1:1, *B*_*N*_ = 0.8*B*), where *H* was the distance between the notch and the root of the cantilever beam. The ratio *K*_*II*_/*K*_*I*_ was less than 5%. The critical specimen thickness limit for plane-strain fracture toughness is given as^12^


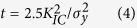


The yield stress *σ*_*y*_ is estimated from Vickers hardness *H*_*V*_ according to Tabor’s approximation 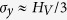. The critical specimen thickness is 1.25 μm for stishovite. We used specimens with the thickness *B* of 20 μm for reliable fracture toughness tests. Furthermore, side-grooves increases the level of constraint, then, promotes a more uniform stress state according to ASTM E399-90.

The stress intensity factor of cantilever beam specimen with side-grooves was calculated, and expressed in the following equation:


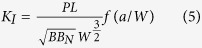


where *P* is the applied force, *L* is the distance between the notch and the loading point, *a* is the crack length, *W* is the cantilever width, *B* is the thickness, and *B*_*N*_ is the minimum thickness measured at the root of the side grooves. The shape factor can be used for 0.2 ≤ *a*/*W* ≤ 0.7:


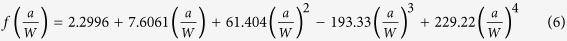


The compliance *C* ≡ *u*/*P* is expressed as:


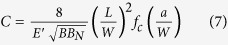



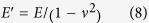


where *E* is Young’s modulus and *v* is Poisson’s ratio.

The dimensionless shape factor for the compliance *f*_*c*_ is given as:


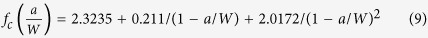


The crack length is expressed as a function of 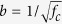:


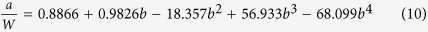


The micro-cantilever beam test is carried out by controlling the displacement *u*′ of the actuator:


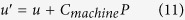


where *u* = *CP* is the displacement of the specimen, and *C*_*machine*_ is the machine compliance. The crack length is determined by the compliance method using equations (7) and (10).

## Additional Information

**How to cite this article**: Yoshida, K. *et al*. Large increase in fracture resistance of stishovite with crack extension less than one micrometer. *Sci. Rep*. **5**, 10993; doi: 10.1038/srep10993 (2015).

## Supplementary Material

Supplementary Information
