# Supplementary material for: Large increase in fracture resistance of stishovite with crack extension less than one micrometer
Source: Sci Rep. 2015 Jun 8;5:10993. doi: 10.1038/srep10993 (PMC4458880; doi:10.1038/srep10993)
Supplement: Supplementary Information [file srep10993-s1.doc]

**Supplementary Information**

**Large increase in fracture resistance of stishovite with crack extension less than one micrometer**

Kimiko Yoshida^1^, Fumihiro Wakai^1^*, Norimasa Nishiyama^2,3^, Risako Sekine^1^, Yutaka Shinoda^1^, Takashi Akatsu^1^, Takashi Nagoshi^4†^ & Masato Sone^4^

1: Secure Materials Center, Materials and Structures Laboratory, Tokyo Institute of Technology, R3-23 4259 Nagatsuta, Midori, Yokohama, 226-8503, Japan

2: Deutsches Elektronen-Synchrotron (DESY), Notkestr. 85, 22607 Hamburg, Germany

3: Precursory Research for Embryonic Science and Technology (PRESTO), Japan Science and Technology Agency (JST), Chiyoda, Tokyo 102-0075, Japan

4: Precision and Intelligence Laboratory, Tokyo Institute of Technology, R2-35 4259 Nagatsuta, Midori, Yokohama, 226-8503, Japan

* Corresponding author, Fumihiro wakai, email: wakai.f.aa@m.titech.ac.jp

^†^present address: Advanced Manufacturing Research Institute, National Institute of Advanced Industrial Science and Technology, 1-2-1 Namiki, Tsukuba, 305-8561, Japan.

**Materials.**

Alumina samples were sintered from commercial α-alumina powder (TM-DAR, Taimei Chemicals Co. Ltd., Japan). As-received powder was heated directly to 1423 K under a uniaxial pressure of 80 MPa using a spark plasma sintering machine (SPS-515S, Fuji Electronic Industrial). Two heating rates were applied; 25 K/min from 873 K to 1273 K, followed by 8 K/min to 1423 K. The density was measured by the Archimedes method. The density of sintered sample was 3.986 g/cm^3^, and the relative density was 100 % using a theoretical density of 3.987 g/cm^3^ for α-alumina. The grain size was determined from TEM micrographs, and evaluated as the equivalent circle diameter. The average grain size was 0.51 μm.

The 3Y-TZP samples were sintered from commercial zirconia powders stabilized with 3 mol% Y_2_O_3_ (TZ3Y, Tosoh Co. Ltd., Japan). As-received powder was heated directly to 1673 K under a uniaxial pressure of 50 MPa using a spark plasma sintering machine. The heating rates was 50 K/min to 1673 K, and holding for 5 min at the temperature. The density of sintered sample was 6.04 g/cm^3^ , and the relative density was 99.8 % using a theoretical density of 6.05 g/cm^3^ for tetragonal zirconia. The average grain size was 0.45 μm.

**Micro-cantilever beam specimens**

The micro-cantilever beam specimens was machined by a focused ion beam (FIB) machining (HITACHI-FB2100) using a high current Ga ion beam (40 keV, 32 nA), followed by fine machining at low currents (6 nA). The specimen size was $H$ = 20 μm, $L$ = 60 μm, $B=W$= 20 μm (Fig. 1a). The cantilevers were notched using an even finer current (300 pA) in the direction perpendicular to the notch length, so that the contamination of Ga ion at the notch root is minimized. The notch tip radius was 50 nm. The side-grooves were cut to guide the crack. The position-marks are made on the top surface and the side surface of the micro-cantilever beam specimens for easy positioning of the indenter (Fig. 1a). The thickness of surface layer damaged by ion beam was estimated by Monte Carlo simulation. For stishovite, the thickness of damage was less than 20 nm by using the grazing 40 keV Ga ion beam.

The exact dimensions of all specimens are given in Table S1. The elastic constants of the materials are also summarized in the table.

Table S1. Dimensions of micro-cantilever beam specimens and elastic constants of materials.

| Materials | E | ν | H | L | W | B | B_N_ | a_0_ |
| --- | --- | --- | --- | --- | --- | --- | --- | --- |
|  | (GPa) |  | (μm) | (μm) | (μm) | (μm) | (μm) | (μm) |
| Stishovite | 530 | 0.205 | 20.1 | 60.6 | 20.8 | 18.4 | 15.0 | 8.2 |
| 3Y-TZP | 214 | 0.309 | 23.1 | 63.4 | 22.3 | 21.6 | 17.3 | 10.7 |
| Alumina | 397 | 0.260 | 20.4 | 61.2 | 19.3 | 18.7 | 15.0 | 9.5 |
| SiO_2_ glass | 72 | 0.17 | 21.0 | 54.0 | 18.8 | 16.2 | － | 9.5 |

Young’s modulus (E), Poisson’s ratio (ν), notch depth (a_0_).

**Crack stability of micro-cantilever beam specimens**

Stable crack propagation is caused by a steep rising R-curve. The crack starts to grow at a critical value $K_{I}=K_{0}$. The crack propagates stably along the R-curve $K_{I}=K_{R}$ untill unstable fracture occurs at the condition^6^:

$\frac{dK_{I}}{da}=\frac{dK_{R}}{da}$. (S1)

The micro-cantilever test is carried out by controlling the displacement $u'$ of the actuator:

$u'=u+C_{machine}P$ (S2)

where $u=CP$ is the displacement of the specimen, and $C_{machine}$ is the machine compliance (0.023 μm/mN). The stress intensity factor is given as a function of crack length:

(S3)

The criterion for the unstable fracture of micro-cantilever beam specimen is given by substituting equation (S3) and equations. (5-8) into equation (S1).
